# Evaluating the effectiveness of different intervention measures for a dengue outbreak in Hangzhou based on a dynamic model

**DOI:** 10.1186/s12889-025-26131-4

**Published:** 2026-01-06

**Authors:** Ling Xu, Rongrong Lu, Haocheng Wu

**Affiliations:** 1 Shangcheng District Center for Disease Control and Prevention (Shangcheng District Health Supervision Institute), Hangzhou, Zhejiang Province 310043 China; 2 Fuyang District Center for Disease Control and Prevention (Fuyang District Health Supervision Institute), Hangzhou, Zhejiang Province 311400 China; 3 Zhejiang Province Center for Disease Control and Prevention, Disease Prevention and Control Innovation Team of Zhejiang Province (2026JKC-04), Hangzhou, Zhejiang Province 310051 China

**Keywords:** Dengue, SEAIR model, Intervention measures, Evaluation

## Abstract

**Background:**

On the basis of 2017 dengue fever outbreak data from Shangcheng District, this study developed a dynamic transmission model to analyze the epidemiological characteristics of dengue fever at a district scale, quantitatively evaluated the effectiveness of different intervention measures, and provided evidence-based support for optimizing outbreak control strategies.

**Methods:**

The outbreak data were obtained from the China Information Network System of Disease Prevention and Control. Some transmission parameters were initially estimated via Berkeley Madonna 8.3.18 software. An SEIAR epidemic model incorporating host-vector bidirectional transmission dynamics was established to evaluate the effectiveness of case isolation, health education, and vector control.

**Results:**

With no intervention, the outbreak would last 225 days, resulting in 8420 cumulative cases, which were both significantly higher than the actual outbreak data (CC = 278 cases, DO = 79 days). Case isolation was the least effective intervention for epidemic control, reducing the cumulative number of cases by only about 71% compared to the estimated incidence without intervention. Vector control was the most effective single intervention. Even a 5% daily vector density reduction intervention could reduce cumulative cases by about 97% and shorten the outbreak duration to 87 days. Increasing the coverage rate and the behavior formation rate of health education could also effectively reduce the number of cumulative cases and shorten the duration of an outbreak. The combined strategy of low-frequency mosquito control (every 3 days) and health education (60% coverage, 50% behavior adoption) and 100% case isolation performed only slightly worse than sustained low-intensity mosquito control alone. However, they were both relatively close to the actual prevention and control effectiveness observed in 2017.

**Conclusions:**

For dengue control in high-density urban areas, we suggested a three-tiered synergistic prevention system: a foundation tier of strict case isolation coupled with intelligent monitoring and early-warning systems; a core tier of sustained high-intensity mosquito control to rapidly suppress vector density during the early epidemic stage; and an optimization tier integrated pulsed mosquito control, health education, and case isolation, thereby addressing the limitations of single interventions and minimizing costs.

## Introduction

Dengue fever (DF) is an acute mosquito-borne infectious disease caused by the dengue virus (DENV), which is endemic across tropical and subtropical regions worldwide, particularly in Southeast Asia, the Americas, the Western Pacific, Africa, and the Eastern Mediterranean [1, 2]. According to the World Health Organization (WHO) report, the cases of DENV infection have increased 30-fold in the past 5 years; consequently, the WHO listed DF as one of the top ten global public health threats in 2019 [2]. Before 1970, DF outbreaks were reported in only nine countries, but now the disease has caused multiple global pandemics and been endemic to more than 100 countries [3–6]. Current estimates indicate that 50–100 million symptomatic infections occur annually, and nearly half of the world’s population lives in a dengue transmission area [7]. The disease imposes considerable health, economic, and political burdens on families and societies [8].

The first dengue outbreak was reported in 1978 in Guangdong Province, Southeast China. Since then, DF has gradually spread from southeastern coastal regions to central and northern provinces [8]. Although China is not considered a dengue-endemic country, surveillance data demonstrate that recent outbreaks are primarily due to indigenous transmission initiated by imported cases. Existing research has identified several factors likely contributing to increasing dengue incidence, including globalization, rapid urbanization, enhanced vector competence, and climate factors such as increased rainfall and elevated temperatures [7, 9].

Located in the Yangtze River Delta region of southeast China, Zhejiang Province has a subtropical monsoon climate that is suitable for the growth of *Aedes albopictus*, the secondary vector of the dengue virus [10]. However, the primary vector, *Aedes aegypti*, has not been found in Zhejiang Province [11]. Moreover, due to high population mobility and strong vector competence, there have been frequent reports of imported dengue cases and occasional local outbreaks. Effective dengue prevention and control require strategies that combine mosquito control, robust surveillance of both the disease and mosquito vectors, vaccination, and community mobilization [12]. However, because of the low seroprevalence rates, the dengue vaccine has not been licensed in China. Therefore, strategies including public awareness, a national reporting system of infectious diseases and public health emergencies, vector control, personal protection, and improved environmental sanitation have greatly reduced dengue prevalence in China [11, 13].

Mathematical models describing dengue fever epidemiological dynamics have been documented as early as 1970 [14], and existing models have been developed to evaluate the effects of cocirculation of multiple strains, the immunological path for disease severity, the impact of vaccination, and the effectiveness of vector control approaches, including host-host transmission models, vector-host transmission models, and within-host models [15, 16]. Additionally, several models have been developed to predict the mosquito population dynamics [17]. Currently, vector control, case and vector surveillance, and health education are commonly implemented for dengue control in China. Previous studies in China and abroad have developed mathematical models to evaluate the effectiveness of vaccination, vector control, case isolation, and awareness programs [18–23]. However, few studies have quantitatively assessed integrated intervention effects at the district level in China, especially using dynamic modeling approaches. In this study, we developed a vector-inclusive transmission dynamics model for a dengue fever outbreak in Shangcheng District, Hangzhou, Zhejiang Province. This model describes the epidemiological characteristics of transmission and assesses the effectiveness of different intervention measures, including case isolation, health education, and vector control, thereby informing evidence-based public health decision making.

## Methods

### Study site

Shangcheng District is located in the southeastern coastal region of China and falls under the jurisdiction of Hangzhou city, Zhejiang Province. It covers an area of 122 square kilometers and administers 14 subdistricts and 201 communities. In 2017, it had a permanent resident population of 1.12 million, with a population density of 9,200 persons per square kilometer. As the central urban district of Hangzhou, Shangcheng District contains several landmark functional zones, most notably transportation mega-hubs (Hangzhou East Station), globally recognized tourism landmarks (West Lake), and modern financial centers. Furthermore, Shangcheng District has a subtropical monsoon climate characterized by mild temperatures and abundant rainfall. Many older residential areas have created typical container-type mosquito breeding environments that were highly suitable for the growth and breeding of *Aedes albopictus*, one of the vectors of the dengue virus. Our monitoring data indicated that the Breteau index for *Aedes albopictus* within the district frequently exceeded the DF transmission risk warning threshold during peak mosquito activity seasons. Given its high population density and frequent population mobility, the district faces a significantly elevated risk of imported DF cases compared with other non-hub urban areas.

### Data source

**Fig. 1:**
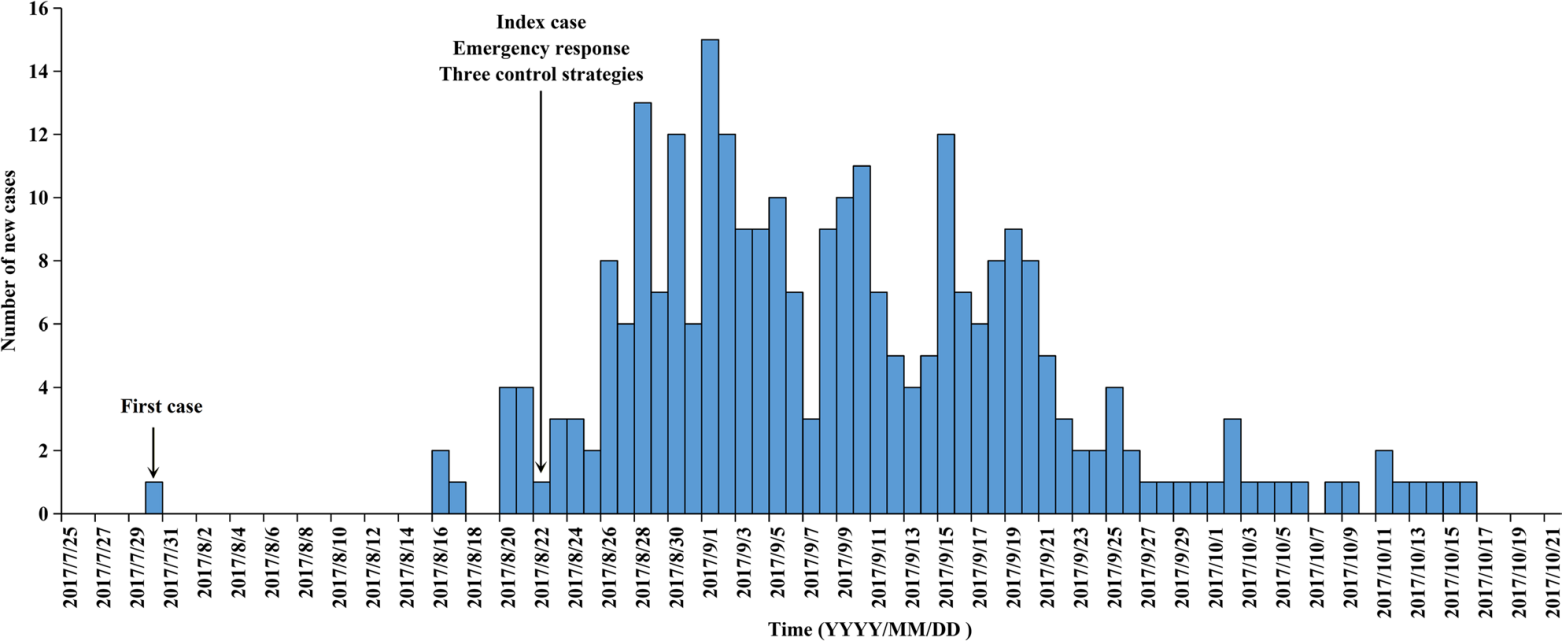
Timeline of the 2017 DF outbreak in Shangcheng district

All individual-level data used in this study were obtained from the China Information System for Disease Control and Prevention. In 2017, a local DF outbreak occurred in Hangzhou. As a densely populated central urban district, Shangcheng District became the epicenter of the outbreak due to its concentration of transportation hubs and complex mosquito breeding sites. The index case, a fitness center employee, developed fever, headache, and diarrhea on August 14, followed by a scattered rash on the limbs on August 15. He sought medical care twice before a laboratory-confirmed DF diagnosis was made on August 22. An epidemiological investigation revealed that the patient had no travel history to dengue-endemic areas within 15 days before symptom onset. This case was presumed to be a secondary infection acquired through local mosquito-borne transmission of an imported virus, with evidence indicating that localized community transmission had been established following intensified case detection. The first patient, who developed symptoms on July 30, was retrospectively identified. The number of cases increased gradually, reaching a daily peak on September 1 and subsequently entering a plateau phase. New cases declined stepwise after September 19, with the last reported case on October 16. The epidemic lasted 79 days in total, yielding a cumulative total of 278 cases (237 laboratory-confirmed and 41 clinically diagnosed cases) without severe or fatal cases. The timeline of this outbreak is shown in Fig. 1. To control the epidemic spread, three core intervention strategies were implemented starting on August 22, including case isolation, vector control, and health education. First, all clinically diagnosed and laboratory-confirmed cases were isolated and treated individually in hospitals until the illness duration exceeded 5 days and fever had subsided for 24 h. Second, local governments immediately conducted district-wide vector control campaigns, including biological, chemical, and environmental measures, to eliminate mosquito-breeding sites and reduce mosquito density. Intervention frequencies were modulated according to risk evaluations. Third, community mobilization and health education initiatives were implemented to disseminate dengue prevention information through multiple channels, including printed materials, health seminars, and social media platforms. According to China’s statutory infectious disease requirements, all cases of dengue fever were reported within 24 h after diagnosis without obvious delay.

### SEIAR model

The present study used the susceptible-exposed-infectious/asymptomatic-recovered (SEIAR) model to simulate the bidirectional human-mosquito transmission. In this model, the total human population (N_h_) was divided into the following five compartments: susceptible individuals (S_h_), exposed individuals (E_h_), infectious individuals (I_h_), asymptomatic individuals (A_h_), and recovered individuals (R_h_). The total female *Aedes* mosquitoes were divided into three compartments: susceptible mosquitoes (S_m_), exposed mosquitoes (E_m_), and infectious mosquitoes (I_m_). The different compartments were interconnected and transitioned according to predefined parameters. The temporal dynamics of each compartment were described by differential equations, forming a system of differential equations that stimulated the DF transmission process.

### Model with no intervention

**Fig. 2:**
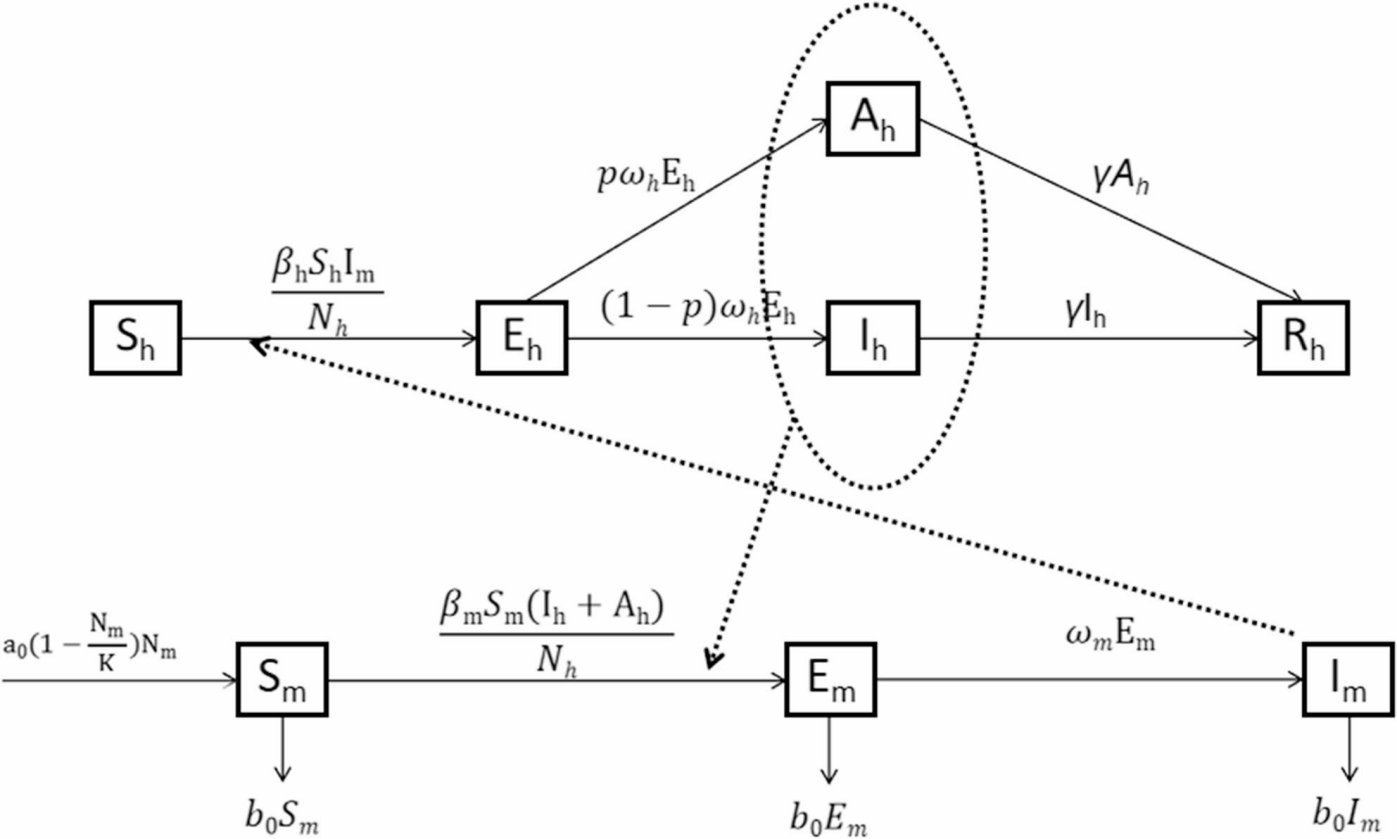
Flowchart of the development of the SEIAR model

The following assumptions should be considered to explain the model construction: population immigration, emigration, and mortality are excluded; both humans and *Aedes* mosquitoes remain universally susceptible to DF; transmission coefficients are identical across dengue virus serotypes; coinfections are not considered; a single exposure confers lifelong immunity with no reinfection or waning immunity; transition rates from susceptible to symptomatic and asymptomatic infectious states are equal; and recovery rates for both symptomatic and asymptomatic infected individuals are equal. The model framework is shown in Fig. 2. The mathematical model with no intervention is expressed as follows:

$$\:\mathrm{d}{\mathrm{S}}_{\mathrm{h}}/\mathrm{d}\mathrm{t}=-\frac{{{\upbeta\:}}_{\mathrm{h}}{\mathrm{S}}_{\mathrm{h}}{\mathrm{I}}_{\mathrm{m}}}{{\mathrm{N}}_{\mathrm{h}}}$$

$$\:\mathrm{d}{\mathrm{E}}_{\mathrm{h}}/\mathrm{d}\mathrm{t}=\frac{{{\upbeta\:}}_{\mathrm{h}}{\mathrm{S}}_{\mathrm{h}}{\mathrm{I}}_{\mathrm{m}}}{{\mathrm{N}}_{\mathrm{h}}}-{{\upomega\:}}_{\mathrm{h}}{\mathrm{E}}_{\mathrm{h}}$$

$$\:\mathrm{d}{\mathrm{I}}_{\mathrm{h}}/\mathrm{d}\mathrm{t}=(1-\mathrm{p}){{\upomega\:}}_{\mathrm{h}}{\mathrm{E}}_{\mathrm{h}}-{\upgamma\:}{\mathrm{I}}_{\mathrm{h}}$$

$$\:\mathrm{d}{\mathrm{A}}_{\mathrm{h}}/\mathrm{d}\mathrm{t}=\mathrm{p}{{\upomega\:}}_{\mathrm{h}}{\mathrm{E}}_{\mathrm{h}}-{\upgamma\:}{\mathrm{A}}_{\mathrm{h}}$$

$$\:\mathrm{d}{\mathrm{R}}_{\mathrm{h}}/\mathrm{d}\mathrm{t}={\upgamma\:}({\mathrm{I}}_{\mathrm{h}}+{\mathrm{A}}_{\mathrm{h}})$$

$$\begin{aligned}\mathrm{d}{\mathrm{S}}_{\mathrm{m}}/\mathrm{d}\mathrm{t}=&{\mathrm{a}}_{0}(1-\frac{{\mathrm{N}}_{\mathrm{m}}}{\mathrm{K}}){\mathrm{N}}_{\mathrm{m}}-{\mathrm{b}}_{0}{\mathrm{S}}_{\mathrm{m}}\\&-\frac{{{\upbeta\:}}_{\mathrm{m}}{\mathrm{S}}_{\mathrm{m}}({\mathrm{I}}_{\mathrm{h}}+{\mathrm{A}}_{\mathrm{h}})}{{\mathrm{N}}_{\mathrm{h}}}\end{aligned}$$

$$\:\mathrm{d}{\mathrm{E}}_{\mathrm{m}}/\mathrm{d}\mathrm{t}=\frac{{{\upbeta\:}}_{\mathrm{m}}{\mathrm{S}}_{\mathrm{m}}({\mathrm{I}}_{\mathrm{h}}+{\mathrm{A}}_{\mathrm{h}})}{{\mathrm{N}}_{\mathrm{h}}}-{\mathrm{b}}_{0}{\mathrm{E}}_{\mathrm{m}}-{{\upomega\:}}_{\mathrm{m}}{\mathrm{E}}_{\mathrm{m}}$$

1 $$\:\mathrm{d}{\mathrm{I}}_{\mathrm{m}}/\mathrm{d}\mathrm{t}={{\upomega\:}}_{\mathrm{m}}{\mathrm{E}}_{\mathrm{m}}-{\mathrm{b}}_{0}{\mathrm{I}}_{\mathrm{m}}$$

### Model with case isolation

In practical epidemic control, we typically implemented mosquito-proof isolation for DF cases to interrupt the human-to-mosquito transmission. However, dengue could spread through both symptomatic and asymptomatic infections, yet in reality, only symptomatic cases could be isolated. Therefore, q was set as the proportion of isolated symptomatic cases, indicating that (1-q) proportion of symptomatic cases and all asymptomatic infections were as the persistent sources of transmission. In this study, q was set in four scenarios, namely, 70%, 80%, 90%, and 100%, and the effects of case isolation were simulated on the 5th, 10th, and 20th day after the first case emerged. The mathematical model is expressed as follows:

$$\:\mathrm{d}{\mathrm{S}}_{\mathrm{h}}/\mathrm{d}\mathrm{t}=-\frac{{{\upbeta\:}}_{\mathrm{h}}{\mathrm{S}}_{\mathrm{h}}{\mathrm{I}}_{\mathrm{m}}}{{\mathrm{N}}_{\mathrm{h}}}$$

$$\:\mathrm{d}{\mathrm{E}}_{\mathrm{h}}/\mathrm{d}\mathrm{t}=\frac{{{\upbeta\:}}_{\mathrm{h}}{\mathrm{S}}_{\mathrm{h}}{\mathrm{I}}_{\mathrm{m}}}{{\mathrm{N}}_{\mathrm{h}}}-{{\upomega\:}}_{\mathrm{h}}{\mathrm{E}}_{\mathrm{h}}$$

$$\:\mathrm{d}{\mathrm{I}}_{\mathrm{h}}/\mathrm{d}\mathrm{t}=(1-\mathrm{p}){{\upomega\:}}_{\mathrm{h}}{\mathrm{E}}_{\mathrm{h}}-{\upgamma\:}{\mathrm{I}}_{\mathrm{h}}$$

$$\:\mathrm{d}{\mathrm{A}}_{\mathrm{h}}/\mathrm{d}\mathrm{t}=\mathrm{p}{{\upomega\:}}_{\mathrm{h}}{\mathrm{E}}_{\mathrm{h}}-{\upgamma\:}{\mathrm{A}}_{\mathrm{h}}$$

$$\:\mathrm{d}{\mathrm{R}}_{\mathrm{h}}/\mathrm{d}\mathrm{t}={\upgamma\:}({\mathrm{I}}_{\mathrm{h}}+{\mathrm{A}}_{\mathrm{h}})$$

$$\begin{aligned}\mathrm{d}{\mathrm{S}}_{\mathrm{m}}/\mathrm{d}\mathrm{t}=&{\mathrm{a}}_{0}(1-\frac{{\mathrm{N}}_{\mathrm{m}}}{\mathrm{K}}){\mathrm{N}}_{\mathrm{m}}-{\mathrm{b}}_{0}{\mathrm{S}}_{\mathrm{m}}\\&-\frac{{{\upbeta\:}}_{\mathrm{m}}{\mathrm{S}}_{\mathrm{m}}\left[\right(1-\mathrm{q}){\mathrm{I}}_{\mathrm{h}}+{\mathrm{A}}_{\mathrm{h}})]}{{\mathrm{N}}_{\mathrm{h}}}\end{aligned}$$

$$\begin{aligned}\mathrm{d}{\mathrm{E}}_{\mathrm{m}}/\mathrm{d}\mathrm{t}=&\frac{{{\upbeta\:}}_{\mathrm{m}}{\mathrm{S}}_{\mathrm{m}}\left[\right(1-\mathrm{q}){\mathrm{I}}_{\mathrm{h}}+{\mathrm{A}}_{\mathrm{h}})]}{{\mathrm{N}}_{\mathrm{h}}}\\&-{\mathrm{b}}_{0}{\mathrm{E}}_{\mathrm{m}}-{{\upomega\:}}_{\mathrm{m}}{\mathrm{E}}_{\mathrm{m}}\end{aligned}$$

2 $$\:\mathrm{d}{\mathrm{I}}_{\mathrm{m}}/\mathrm{d}\mathrm{t}={{\upomega\:}}_{\mathrm{m}}{\mathrm{E}}_{\mathrm{m}}-{\mathrm{b}}_{0}{\mathrm{I}}_{\mathrm{m}}$$

### Model with health education

Knowledge, attitudes, and practices remained the most effective tools for dengue prevention and control [24]. Health education could reduce vector-borne transmission efficiency by promoting protective behaviors in the population. The intervention efficacy was determined by the product of three parameters: coverage rate (C), behavior adoption rate (E), and repellent efficacy (F). This model was expressed through adjusted transmission coefficients for both human-to-mosquito (βₘ) and mosquito-to-human (βₕ) infection rates. According to previous studies, the protective efficacy of various repellents against *Aedes albopictus* ranges between 76.3% and 97.8% [25], with a median value of 88.8% adopted for modeling; the behavior adoption rate varies from 24.2% to 75% [26, 27], with a median value of 50% as the baseline scenario parameter. We established four coverage scenarios (C = 50%, 60%, 70%, 80%) with fixed behavior adoption rate (E = 50%) and repellent efficacy (F = 88.8%) to investigate how health education coverage affects dengue transmission dynamics. By holding coverage constant (C = 60%) and maintaining prevention effectiveness (F = 88.8%), we subsequently evaluated the marginal effects of knowledge conversion efficiency by testing five behavior adoption rates (E = 30%, 40%, 50%, 60%, 70%). The model with health education is expressed as follows:

$$\:\mathrm{d}{\mathrm{S}}_{\mathrm{h}}/\mathrm{d}\mathrm{t}=-\frac{{(1-\mathrm{C}\ast\:\mathrm{E}\ast\:F){\upbeta\:}}_{\mathrm{h}}{\mathrm{S}}_{\mathrm{h}}{\mathrm{I}}_{\mathrm{m}})}{{\mathrm{N}}_{\mathrm{h}}}$$

$$\:\mathrm{d}{\mathrm{E}}_{\mathrm{h}}/\mathrm{d}\mathrm{t}=\frac{{(1-\mathrm{C}\ast\:\mathrm{E}\ast\:F){\upbeta\:}}_{\mathrm{h}}{\mathrm{S}}_{\mathrm{h}}{\mathrm{I}}_{\mathrm{m}}}{{\mathrm{N}}_{\mathrm{h}}}-{{\upomega\:}}_{\mathrm{h}}{\mathrm{E}}_{\mathrm{h}}$$

$$\:\mathrm{d}{\mathrm{I}}_{\mathrm{h}}/\mathrm{d}\mathrm{t}=(1-\mathrm{p}){{\upomega\:}}_{\mathrm{h}}{\mathrm{E}}_{\mathrm{h}}-{\upgamma\:}{\mathrm{I}}_{\mathrm{h}}$$

$$\:\mathrm{d}{\mathrm{A}}_{\mathrm{h}}/\mathrm{d}\mathrm{t}=\mathrm{p}{{\upomega\:}}_{\mathrm{h}}{\mathrm{E}}_{\mathrm{h}}-{\upgamma\:}{\mathrm{A}}_{\mathrm{h}}$$

$$\:\mathrm{d}{\mathrm{R}}_{\mathrm{h}}/\mathrm{d}\mathrm{t}={\upgamma\:}({\mathrm{I}}_{\mathrm{h}}+{\mathrm{A}}_{\mathrm{h}})$$

$$\begin{aligned}\mathrm{d}{\mathrm{S}}_{\mathrm{m}}/\mathrm{d}\mathrm{t}=&{\mathrm{a}}_{0}(1-\frac{{\mathrm{N}}_{\mathrm{m}}}{\mathrm{K}}){\mathrm{N}}_{\mathrm{m}}-{\mathrm{b}}_{0}{\mathrm{S}}_{\mathrm{m}}\\&-\frac{{(1-\mathrm{C}\ast\:\mathrm{E}\ast\:F){\upbeta\:}}_{\mathrm{m}}{\mathrm{S}}_{\mathrm{m}}({\mathrm{I}}_{\mathrm{h}}+{\mathrm{A}}_{\mathrm{h}})}{{\mathrm{N}}_{\mathrm{h}}}\end{aligned}$$

$$\begin{aligned}\mathrm{d}{\mathrm{E}}_{\mathrm{m}}/\mathrm{d}\mathrm{t}=&\frac{{(1-\mathrm{C}\ast\:\mathrm{E}\ast\:F){\upbeta\:}}_{\mathrm{m}}{\mathrm{S}}_{\mathrm{m}}({\mathrm{I}}_{\mathrm{h}}+{\mathrm{A}}_{\mathrm{h}})}{{\mathrm{N}}_{\mathrm{h}}}\\&-{\mathrm{b}}_{0}{\mathrm{E}}_{\mathrm{m}}-{{\upomega\:}}_{\mathrm{m}}{\mathrm{E}}_{\mathrm{m}}\end{aligned}$$

3 $$\:\mathrm{d}{\mathrm{I}}_{\mathrm{m}}/\mathrm{d}\mathrm{t}={{\upomega\:}}_{\mathrm{m}}{\mathrm{E}}_{\mathrm{m}}-{\mathrm{b}}_{0}{\mathrm{I}}_{\mathrm{m}}$$

### Model with vector control

Vector control was a core strategy for dengue prevention in China and involved environmental management, chemical insecticides, and biological control [28]. We simulated the effects of continuous and pulsed vector control interventions, separately.

Continuous vector control meant that all mosquito compartments undergo a daily reduction instantaneously according to predetermined intervention proportions. To achieve dynamic simulation of the *Aedes* mosquito population, we assumed x as an additional mosquito mortality representing the combined effect of chemical mosquito control and breeding site management. Therefore, vector control interventions disrupted the mosquito population equilibrium by elevating mortality, with the total mortality rate defined as the sum of the natural mortality rate (b_0_) and the additional mortality rate (x) induced by the interventions. In real-world scenarios, mosquito populations were not eradicated immediately after control implementation. Instead, a progressive density decline occured post-intervention, with populations subsequently persisting at significantly reduced equilibrium levels. To simulate real-world scenarios that integrated rapid initial suppression of adult mosquitoes with sustained breeding source elimination, we incorporated the natural constant *e* to model mosquito population dynamics through exponential decay kinetics. On the basis of actual efficacy, x was set in three scenarios, namely, x = ln(0.95), x = ln(0.90), and x = ln(0.85), which represent a 5% daily reduction in mosquito density at day t, a 10% daily reduction in mosquito density at day t, and a 15% daily reduction in mosquito density at day t, respectively. The model with continuous vector control is expressed as follows:

$$\:\mathrm{d}{\mathrm{S}}_{\mathrm{h}}/\mathrm{d}\mathrm{t}=-\frac{{{\upbeta\:}}_{\mathrm{h}}{\mathrm{S}}_{\mathrm{h}}{\mathrm{I}}_{\mathrm{m}}}{{\mathrm{N}}_{\mathrm{h}}}$$

$$\:\mathrm{d}{\mathrm{E}}_{\mathrm{h}}/\mathrm{d}\mathrm{t}=\frac{{{\upbeta\:}}_{\mathrm{h}}{\mathrm{S}}_{\mathrm{h}}{\mathrm{I}}_{\mathrm{m}}}{{\mathrm{N}}_{\mathrm{h}}}-{{\upomega\:}}_{\mathrm{h}}{\mathrm{E}}_{\mathrm{h}}$$

$$\:\mathrm{d}{\mathrm{I}}_{\mathrm{h}}/\mathrm{d}\mathrm{t}=(1-\mathrm{p}){{\upomega\:}}_{\mathrm{h}}{\mathrm{E}}_{\mathrm{h}}-{\upgamma\:}{\mathrm{I}}_{\mathrm{h}}$$

$$\:\mathrm{d}{\mathrm{A}}_{\mathrm{h}}/\mathrm{d}\mathrm{t}=\mathrm{p}{{\upomega\:}}_{\mathrm{h}}{\mathrm{E}}_{\mathrm{h}}-{\upgamma\:}{\mathrm{A}}_{\mathrm{h}}$$

$$\:\mathrm{d}{\mathrm{R}}_{\mathrm{h}}/\mathrm{d}\mathrm{t}={\upgamma\:}({\mathrm{I}}_{\mathrm{h}}+{\mathrm{A}}_{\mathrm{h}})$$

$$\begin{aligned}\mathrm{d}{\mathrm{S}}_{\mathrm{m}}/\mathrm{d}\mathrm{t}=&{\mathrm{a}}_{0}(1-\frac{{\mathrm{N}}_{\mathrm{m}}}{\mathrm{K}}){\mathrm{N}}_{\mathrm{m}}-({\mathrm{b}}_{0}+\mathrm{x}){\mathrm{S}}_{\mathrm{m}}\\&-\frac{{{\upbeta\:}}_{\mathrm{m}}{\mathrm{S}}_{\mathrm{m}}({\mathrm{I}}_{\mathrm{h}}+{\mathrm{A}}_{\mathrm{h}})}{{\mathrm{N}}_{\mathrm{h}}}\end{aligned}$$

$$\begin{aligned}\mathrm{d}{\mathrm{E}}_{\mathrm{m}}/\mathrm{d}\mathrm{t}=&\frac{{{\upbeta\:}}_{\mathrm{m}}{\mathrm{S}}_{\mathrm{m}}({\mathrm{I}}_{\mathrm{h}}+{\mathrm{A}}_{\mathrm{h}})}{{\mathrm{N}}_{\mathrm{h}}}\\&-({\mathrm{b}}_{0}+\mathrm{x}){\mathrm{E}}_{\mathrm{m}}-{{\upomega\:}}_{\mathrm{m}}{\mathrm{E}}_{\mathrm{m}}\end{aligned}$$

4 $$\:\mathrm{d}{\mathrm{I}}_{\mathrm{m}}/\mathrm{d}\mathrm{t}={{\upomega\:}}_{\mathrm{m}}{\mathrm{E}}_{\mathrm{m}}-({\mathrm{b}}_{0}+\mathrm{x}){\mathrm{I}}_{\mathrm{m}}$$

In the pulsed vector control, all mosquito compartments were instantaneously reduced by specified intervention proportions on treatment days and follow natural birth-death dynamics on non-treatment days. Assuming a pulsed mosquito control frequency of once every three days, with an instantaneous daily mosquito density reduction of 5% per intervention, the mosquito population in each compartment (S_m_, E_m_, I_m_) was directly multiplied by 95% on each implementation day (e.g., at t_x_= t_start_, t_start+3_, t_start+6_, …). The model with pulsed vector control is expressed as follows:

$$\:\mathrm{d}{\mathrm{S}}_{\mathrm{h}}/\mathrm{d}\mathrm{t}=-\frac{{{\upbeta\:}}_{\mathrm{h}}{\mathrm{S}}_{\mathrm{h}}{\mathrm{I}}_{\mathrm{m}}}{{\mathrm{N}}_{\mathrm{h}}}$$

$$\:\mathrm{d}{\mathrm{E}}_{\mathrm{h}}/\mathrm{d}\mathrm{t}=\frac{{{\upbeta\:}}_{\mathrm{h}}{\mathrm{S}}_{\mathrm{h}}{\mathrm{I}}_{\mathrm{m}}}{{\mathrm{N}}_{\mathrm{h}}}-{{\upomega\:}}_{\mathrm{h}}{\mathrm{E}}_{\mathrm{h}}$$

$$\:\mathrm{d}{\mathrm{I}}_{\mathrm{h}}/\mathrm{d}\mathrm{t}=(1-\mathrm{p}){{\upomega\:}}_{\mathrm{h}}{\mathrm{E}}_{\mathrm{h}}-{\upgamma\:}{\mathrm{I}}_{\mathrm{h}}$$

$$\:\mathrm{d}{\mathrm{A}}_{\mathrm{h}}/\mathrm{d}\mathrm{t}=\mathrm{p}{{\upomega\:}}_{\mathrm{h}}{\mathrm{E}}_{\mathrm{h}}-{\upgamma\:}{\mathrm{A}}_{\mathrm{h}}$$

$$\:\mathrm{d}{\mathrm{R}}_{\mathrm{h}}/\mathrm{d}\mathrm{t}={\upgamma\:}({\mathrm{I}}_{\mathrm{h}}+{\mathrm{A}}_{\mathrm{h}})$$

$$\begin{aligned}\mathrm{d}{\mathrm{S}}_{\mathrm{m}}/\mathrm{d}\mathrm{t}=&{\mathrm{a}}_{0}(1-\frac{{\mathrm{N}}_{\mathrm{m}}}{\mathrm{K}}){\mathrm{N}}_{\mathrm{m}}-({\mathrm{b}}_{0}+{\mathrm{t}}_{\mathrm{x}}){\mathrm{S}}_{\mathrm{m}}\\&-\frac{{{\upbeta\:}}_{\mathrm{m}}{\mathrm{S}}_{\mathrm{m}}({\mathrm{I}}_{\mathrm{h}}+{\mathrm{A}}_{\mathrm{h}})}{{\mathrm{N}}_{\mathrm{h}}}\end{aligned}$$

$$\begin{aligned}\mathrm{d}{\mathrm{E}}_{\mathrm{m}}/\mathrm{d}\mathrm{t}=&\frac{{{\upbeta\:}}_{\mathrm{m}}{\mathrm{S}}_{\mathrm{m}}({\mathrm{I}}_{\mathrm{h}}+{\mathrm{A}}_{\mathrm{h}})}{{\mathrm{N}}_{\mathrm{h}}}\\&-({\mathrm{b}}_{0}+{\mathrm{t}}_{\mathrm{x}}){\mathrm{E}}_{\mathrm{m}}-{{\upomega\:}}_{\mathrm{m}}{\mathrm{E}}_{\mathrm{m}}\end{aligned}$$

5 $$\:\mathrm{d}{\mathrm{I}}_{\mathrm{m}}/\mathrm{d}\mathrm{t}={{\upomega\:}}_{\mathrm{m}}{\mathrm{E}}_{\mathrm{m}}-({\mathrm{b}}_{0}+{\mathrm{t}}_{\mathrm{x}}){\mathrm{I}}_{\mathrm{m}}$$

### Estimation of parameters

The models included 27 parameters, as shown in Table 1. The total human population was reported from the system. Seven variables, S_m0_, E_m0_, I_m0_, β_h_, β_m_, a_0_, and K, were estimated within a framework that utilized the fourth-order Runge-Kutta method for numerical simulation and the coefficient of determination (*R²*) for assessing modeling accuracy. Another five parameters, including p, $\:\mathrm{ω}$_h_, $\:\mathrm{ω}$_m_, $\:\mathrm{{\gamma}}$, and b_0_, were obtained from related studies [29–32]. The proportion of asymptomatic infections is 75%, thus *p* = 0.75. The mean incubation period for DF infection is 5 days, thus $\:\mathrm{ω}$_h_=0.2. After a blood meal is taken from an infected person, the virus requires 8–12 days (extrinsic incubation period) in mosquitoes before it can be transmitted to another human. Here, we simulated 10 days in our model, thus $\:\mathrm{ω}$_m_ = 0.1. The infectious period of DF patients ranges from 1 day prior to symptom onset until 5 days after symptoms appear, with vector-control isolation measures generally lifted after day 5 of illness, thus $\:\mathrm{{\gamma}}$=0.2. The average lifespan of Aedes mosquitoes is 21 days, thus b_0_ = 1/21. The reduction rate of mosquitoes due to vector control measures (x) was set to -ln(0.95), -ln(0.90) and -ln(0.85), which corresponded to a 5% daily reduction in mosquito density, a 10% daily reduction in mosquito density and a 15% daily reduction in mosquito density, respectively. The proportion of case isolation (q) was set to 100%, which meant that every symptomatic case should be isolated in time.

**Table 1:** Description and values of the parameters in the SEIAR model

Parameter	Descriptions	Unit	Value	Source
S_h_	Number of susceptible humans	person	-	-
E_h_	Number of exposed humans	person	-	-
I_h_	Number of infectious humans	person	-	-
A_h_	Number of asymptomatic humans	person	-	-
R_h_	Number of recovered humans	person	-	-
S_m_	Number of susceptible mosquitoes	mosquito	-	-
E_m_	Number of exposed mosquitoes	mosquito	-	-
I_m_	Number of infectious mosquitoes	mosquito	-	-
N_h_	Total human population size	person	1,120,000	Reported
N_v_	Total mosquito population size	mosquito	-	-
S_m0_	Initial values of susceptible mosquitoes		47,921,500	Curve fitting
E_m0_	Initial values of exposed mosquitoes		1	Curve fitting
I_m0_	Initial values of infectious mosquitoes		1	Curve fitting
$\:\mathrm{{\beta}}$_h_	Transmission rate from an infected mosquito to a susceptible human		0.0266295	Curve fitting
$\:\mathrm{{\beta}}$_m_	Transmission rate from an infected human to a susceptible mosquito		0.769698	Curve fitting
$\:\mathrm{p}$	Proportion of human asymptomatic infection		0.75	Reference[29]
$\:\mathrm{ω}$_h_	Changing rate of humans from the exposed state to the symptomatic and asymptomatic state		0.2	Reference[30, 31]
$\:\mathrm{ω}$_m_	Changing rate of mosquitoes from the exposed state to the infectious state		0.1	Reference[30, 31]
$\:\mathrm{{\gamma}}$	Recovery rate of human from the symptomatic state to the recovered state		0.2	Reference[32]
a_0_	Natural growth rate of mosquitoes		0.00000135207	Curve fitting
b_0_	Density-independent death rate		1/21	Reference[30, 31]
C	Coverage rates of health education		-	Setting
E	Behavior formation rates of health education		-	Setting
F	Efficacy of repellents against mosquito		0.888	Reference[25]
x	Reduction rate of mosquitoes due to vector control measures		-	Setting
K	Carrying capacity of mosquitoes	1	8,294,190	Curve fitting
q	Proportion of case isolation	day^− 1^	-	Setting

### Sensitivity analysis

Since five parameters (p, $\:\mathrm{ω}$_h_, $\:\mathrm{ω}$_m_, $\:\mathrm{{\gamma}}$, b_0_) were adapted from the literature, uncertainty might exist for the simulation. Thus, sensitivity analysis was performed by splitting each parameter into 1,000 values. The value ranges of the five parameters were 0–1, 0.14–0.25 (4–7 days), 0.07–0.14 (7–14 days), 0.08–0.25 (4–12 days), 0.02–0.13 (8–42 days), respectively.

### Simulation steps and statistical analysis

On the basis of the implementation timeline of the control measures, the outbreaks were divided into two periods. The period from July 30 to August 22 without any interventions was the baseline stage and was used for model parameter calibration. The period after August 22 was the intervention stage, during which comprehensive measures such as health education, case isolation, and vector control were implemented to evaluate the intervention efficacy. The modeling process in this study consisted of three steps.

Step 1: We simulated the epidemic without any interventions based on the data of the baseline stage and obtained parameters S_m0_, E_m0_, I_m0_, β_h_, β_m_, a_0_, and K. Berkeley Madonna 8.3.18 (University of California at Berkeley, Berkeley, USA) were employed for model simulation. The Runge–Kutta method of order four with the tolerance set at 0.001 was used to perform curve fitting.

Step 2: The coefficient of determination test was performed to judge the goodness of fit of curve fitting between the simulation models and the reported data, which was assessed by SPSS 23.0 (IBM Corp, Armonk, NY, USA). Statistical significance was indicated by *R*^*2*^ ≥ 0.5 and *P* < 0.05, and the model fit was good.

Step 3: We estimated the effectiveness of different interventions, including health education with different coverage rates and behavior formation rates, case isolation with different proportions, vector control with different frequencies and different levels of daily reduction in mosquito density. The cumulative cases (CC) and duration of outbreaks (DO) were used to quantitatively evaluate the effects of different intervention measures.

## Results

### Model fitting and sensitivity analysis

The simulation results revealed that the present model fit well (*R*^*2*^ = 0.896, F = 146.467, *P* < 0.05) with the reported epidemic curve when S_m0_ was 47,921,500, E_m0_ was 1, I_m0_ was 1, β_h_ was 0.0266295, β_m_ was 0.769698, a_0_ was 0.00000135207 and K was 8,294,190.

**Fig. 3:**
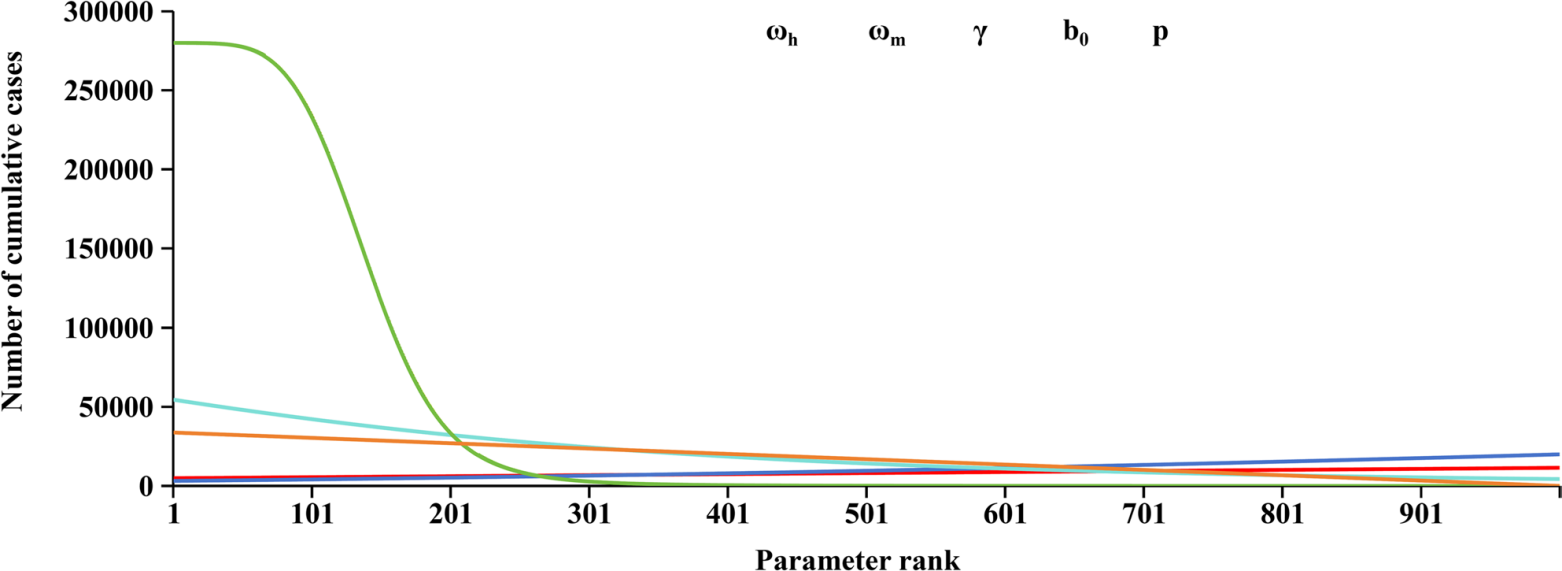
Sensitivity analysis of the ω_h_, ω_m_, γ, b_0_, and p parameters based on the outbreak

The results of the sensitivity analysis revealed that the model was insensitive to the parameters $\:\mathrm{ω}$_h_, $\:\mathrm{ω}$_m_, γ and p but was sensitive to b_0_ (Fig. 3). When b_0_ increased from 0.02 (corresponding to a 42-day mosquito lifespan) to 0.13 (corresponding to an 8-day mosquito lifespan), CC exhibited an exponential decline as the mosquito lifespan decreased. Increased b_0_ values shorten the lifespan of infected mosquitoes, thereby reducing the effective transmission window between humans and mosquitoes, and ultimately impairing the ability of pathogen to sustain transmission within the mosquito population.

### Effectiveness of interventions

In the baseline scenario without intervention, the model predicted that the DF epidemic would follow an exponential growth trend, with daily new cases peaking on November 1st (110 cases), and the CC would rise continuously, ultimately reaching 8420 cases, with the DO of 225 days.

**Fig. 4:**
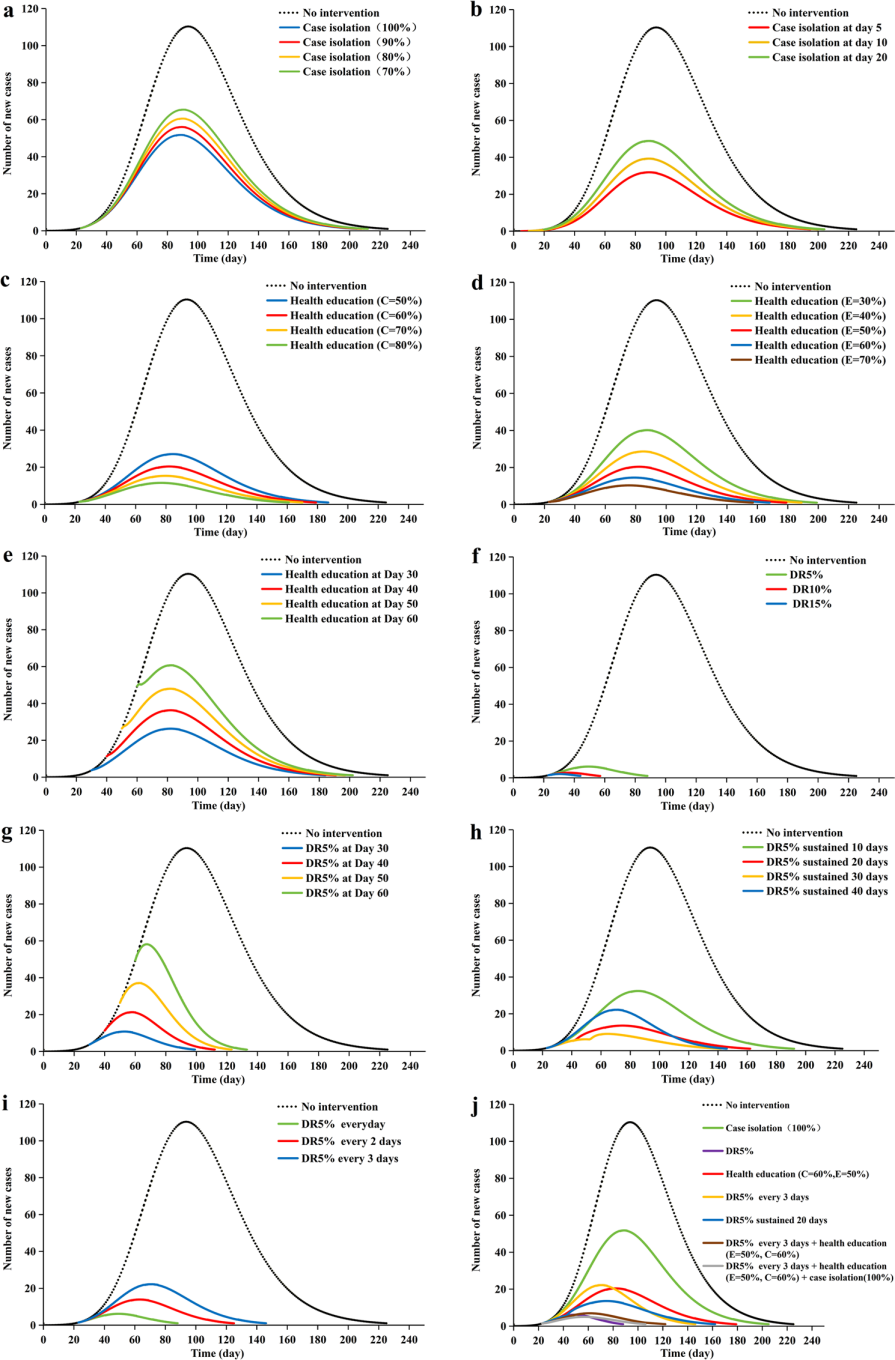
Effectiveness of different interventions on DF outbreaks. (**a**) The effects of different proportions of case isolation. The starting time of the case isolation was August 22. (**b**) Effects of different initial times of implementing case isolation. (**c**) Effects of different coverage rates of health education when behavior adoption rate is fixed at 50%. The starting time of health education was August 22. (**d**) Effects of different behavior adoption rates of health education when coverage rate is fixed at 60%. The starting time of health education was August 22. (**e**) Effects of different initial times of health education when behavior adoption rate is fixed at 50% and overage rate is fixed at 60%. (**f**) Effects of different levels of daily reduction in mosquito density induced by vector control. The starting time of vector control was August 22. (**g**) Effects of different initial times of 5% daily reduction in mosquito density induced by vector control. (**h**) Effects of duration of 5% daily reduction in mosquito density induced by vector control. The starting time of vector control was August 22. (**i**) Effects of different implementation schedules of 5% daily reduction in mosquito density induced by vector control. The starting time of vector control was August 22. (**j**) Comparisons of the effects of implementing single and combined interventions. The vertical axis indicates the number of new cases

Our results indicated that case isolation was not satisfactory for controlling the development of the epidemic. Implementing case isolation at higher coverage levels (70–100%) 23 days after the onset of the first case into the outbreak resulted in moderate reductions (40–53%) in cumulative cases and a slight shortening of the outbreak duration (Fig. 4a; Table 2). However, initiating 100% isolation much earlier (e.g., by day 5) only increased the reduction in cases to approximately 71% (Fig. 4b; Table 2).

**Table 2:** Evaluation of the different intervention measures on the DF outbreak

Intervention	CC (cases)	% decrease of CC	DO (days)	Reduced days of DO (days)
Actual outbreak data	278	-	79	-
No intervention	8420	-	225	-
Case isolation				
70%	5018	40.40	211	14
80%	4649	44.79	209	16
90%	4302	48.91	207	18
100%	3974	52.80	204	21
100% at Day 5	2447	70.94	194	31
100% at Day 10	3017	64.17	199	26
100% at Day 20	3752	55.44	203	22
Health education				
E = 50%, C = 50%	2074	75.37	186	39
E = 50%, C = 60%	1559	81.48	178	47
E = 50%, C = 70%	1169	86.12	169	56
E = 50%, C = 80%	875	89.61	160	65
E = 30%, C = 60%	3082	63.40	198	27
E = 40%, C = 60%	2196	73.92	188	37
E = 50%, C = 60%	1559	81.48	178	47
E = 60%, C = 60%	1103	86.90	167	58
E = 70%, C = 60%	779	90.75	156	69
E = 50%, C = 60% at Day 30	2010	76.13	183	42
E = 50%, C = 60% at Day 40	2763	67.19	190	35
E = 50%, C = 60% at Day 50	3614	57.08	196	29
E = 50%, C = 60% at Day 60	4504	46.51	201	24
Continuous vector control				
DR5%	262	96.89	87	138
DR10%	83	99.01	56	169
DR15%	46	99.45	43	182
DR5% at Day 30	469	94.43	98	127
DR5% at Day 40	932	88.93	111	114
DR5% at Day 50	1619	80.77	122	103
DR5% at Day 60	2506	70.23	132	93
DR5% sustained 10 days	2482	70.52	191	34
DR5% sustained 20 days	1018	87.91	161	64
DR5% sustained 30 days	606	92.80	141	84
DR5% sustained 40 days	450	94.66	128	97
Pulsed vector control				
DR5% everyday	262	96.89	87	138
DR5% every 2 days	760	90.97	124	101
DR5% every 3 days	1339	84.10	145	80
Combinations				
DR5% every 3 days + health education (E = 50%, C = 60%)	416	95.06	122	103
DR5% every 3 days + health education (E = 50%, C = 60%) + 100% case isolation	285	96.62	106	119

*CC* the cumulative cases, *DO* duration of outbreak, *E* behavior adoption rate of health education campaigns, *C* coverage rate of health education campaigns, *DR5%* 5% daily reduction in mosquito density reduced by vector control, *DR10%* 10% daily reduction in mosquito density reduced by vector control, *DR15%* 15% daily reduction in mosquito density reduced by vector control

The model revealed that increasing the coverage and behavior adoption rate of awareness campaigns could reduce the CC and shorten the DO. With behavior adoption fixed at 50%, increasing coverage from 50% to 80% reduced CC by over 14% points (from 75.4% to 89.6% lower than baseline) and shortened DO by 26 days (from 186 to 160 days) (Fig. 4c; Table 2). With coverage fixed at 60%, increasing the adoption rate from 30% to 70% more than doubled the reduction in CC (from 63.4% to 90.8%) and shortened DO by 42 days (from 198 to 156 days) (Fig. 4d; Table 2). Furthermore, the earlier health education was implemented, the better the control effect. As the intervention start date was delayed from day 30 to day 60, with coverage and adoption rates fixed at 60% and 50% respectively, the reduction in CC decreased from over 76% to under 47%, and the corresponding DO increased from 183 to 201 days (Fig. 4e; Table 2).

The model indicated that a reduction in mosquito density could decrease the CC and shorten the DO, and the greater the daily reduction in mosquito density was, the lower the magnitude of the epidemic. First, we implemented daily mosquito density reductions of 5%, 10%, and 15%, abbreviated as DR5%, DR10%, and DR15%, respectively. Under these three scenarios, the CC declined to 262, 83, and 46 cases, which were 96.89%, 99.01%, and 99.45% lower than those in the non-intervention scenario. The DO was shortened to 87 days, 56 days, and 43 days, respectively (Fig. 4f; Table 2). Second, we estimated the effectiveness of different initial times of implementing vector control, setting the daily reduction in mosquito density at 5%. Earlier implementation was critical. Delaying the start of a 5% daily reduction measure from day 30 to day 60 resulted in a progressive and marked decline in effectiveness, with CC reductions falling from approximately 94% to 70% and DO decreasing from 127 to 93 days (Fig. 4g; Table 2). Implementing pulsed vector control measures also significantly enhanced epidemic control efficacy. When vector control was implemented every day, every other day and every three days with 5% daily reduction in mosquito density, the CC was 262, 760, and 1339 cases, respectively. Surprisingly, even when vector control was implemented every three days, the CC still decreased over 84% compared with the estimated incidence without intervention. Furthermore, continuous mosquito control measures were essential. Even when the DR5% vector control intervention was applied for only 20 days, both CC (1018 cases) and DO (161 days) were lower than those of the pulsed vector control measures implemented every 3 days (Fig. 4h and i; Table 2).

The effects of single control showed that vector control measures had significant advantages in reducing the CC and shortening the DO. Even a low-intensity (5% daily reduction) continuous vector control strategy outperformed 100% case isolation, achieving a reduction in CC of over 93% (262 vs. 3974 cases) and a shortening of DO by over 57% (87 vs. 204 days). Compared with continuous vector control at DR5%, health education (E = 50%, C = 60%) campaigns demonstrated significantly reduced effectiveness, yielding higher CC (1559 vs. 262 cases) and longer DO (178 vs. 87 days). Nevertheless, it was marginally inferior to both the pulsed vector control every 3 days (CC = 1339 cases, DO = 145 days) and the 20-day sustained mosquito intervention (CC = 1018, DO = 161 days), thus reflecting the cost-effectiveness advantages of the behavioral interventions. The results of comprehensive control showed that the combination of pulsed vector control (every 3 days), health education (E = 50%, C = 60%), and 100% case isolation resulted in a CC of 285 cases and a DO of 106 days. This outcome was superior to that achieved with pulsed vector control and health education alone (CC = 416 cases, DO = 122 days), but slightly less effective than continuous low-intensity vector control alone (CC = 262 cases, DO = 87 days). (Fig. 4j; Table 2).

## Discussion

In this study, SEIAR models were established based on the DF outbreak data from Shangcheng District (Hangzhou, China) in 2017. The results of the coefficient of determination (*R*^*2*^ ≥ 0.5) and significance test (*P* < 0.05) revealed that the model had a good fit with the actual epidemic without intervention, confirming its applicability for simulating outbreaks at the sub-district level and evaluating the effectiveness of interventions. The model predicted that without any interventions, the CC would have reached 8420. In the actual epidemic, however, the final number of reported cases was 278 due to the implementation of comprehensive interventions such as health education, case isolation, and emergency mosquito control, representing a 96.70% reduction compared with the predicted value. These findings provided evidence that the practical control measures used in this outbreak were highly effective.

Although case isolation is a classical measure used to prevent and control infectious disease outbreaks [33], it is not the primary intervention used during DF outbreak. Our model indicated that even with 100% case isolation, the CC remained as high as 2447, representing a 70.94% reduction compared with the no-intervention scenario. This result was consistent with previous research [18, 19]. On the one hand, approximately 75% of asymptomatic cases remained undetected due to the absence of clinical symptoms, indicating this population was not isolated and served as a hidden source of infection, continuously excreting viruses to mosquito vectors. On the other hand, our study revealed an 8-day interval between symptom onset and diagnosis in index cases, during which the virus had already established community transmission chains through mosquito bites, thus case isolation alone was insufficient to block the infected-mosquito-to-human transmission pathway. Following the COVID-19 pandemic, national infectious disease intelligent monitoring and early warning front-end software has been designed and developed in China. This system can realize the major transformation of infectious disease monitoring from traditional passive reporting to modern active perception, providing technological support for shortening the time interval between symptom onset, clinical diagnosis, and case isolation [34]. Additionally, serological screening of asymptomatic infections must be implemented when necessary, integrated with early-stage vector control guided by mosquito density surveillance, to establish a comprehensive containment chain enabling bidirectional human-mosquito transmission interruption.

Parameter sensitivity analysis revealed that the natural mortality rate of *Aedes* mosquitoes (b_0_) was a pivotal model parameter. When b_0_ increased from 0.02 to 0.13, the CC exhibited an exponential decline. This phenomenon theoretically validated the core role of vector control in epidemic containment systems. Our model demonstrated that the DR5% continuous vector control yielded 262 cumulative cases, equivalent to only 6.59% of the CC of 100% case isolation and representing an 83.19% reduction compared with health education (E = 50%, C = 60%). Vector control could reduce contact between vectors and hosts, thereby interrupting dengue virus transmission, which was consistent with the finding of previous studies [35, 36]. Notably, at a 5% daily mosquito density reduction level, 20-day sustained vector control reduced 321 cases compared with every-3-day pulsed intervention mode, indicating that early continuous suppression was critical for disrupting the transmission cycle between humans and mosquitoes.

Although the efficacy of health education (E = 50%, C = 60%) was substantially lower than that of continuous 5% daily density reduction, it approached the level achieved by the every-3-day pulsed control or 20-day sustained suppression. The knowledge and management strategies of communities are crucial for mitigating and controlling the threat of arboviral diseases [37], and health education can reduce dengue transmission risk by enhancing community awareness of preventive measures, and promoting the adoption of insect repellents, bed nets, and breeding site elimination [38, 39]. The combined strategy of every-3-day pulsed control and health education (E = 50%, C = 60%) demonstrated comparable efficacy to that of the DR5% continuous vector control. This suggested that in resource-limited scenarios, this integrated approach might represent a cost-effective alternative. The effectiveness of health education in prevention and control is determined by the coverage rate and behavior formation rate. Previous studies have noted that there are differences in the conversion between knowledge acquisition and behavior change across different regions and populations [40–42]. With the popularization of digital communication methods, increasing the coverage rate of health education is no longer a difficult task, while improving the behavior formation rate has become the main challenge in optimizing the effectiveness of health education at present. In this study, simulation data revealed that with a coverage rate of 60%, increasing the behavior formation rate from 50% to 70% could reduce the CC by 780, while decreasing it to 30% would result in an additional 1523 cases. This quantitative result indicated that simply expanding the coverage of health education was insufficient to achieve the desired prevention and control effects. It will be essential to develop targeted health education programs that account for differences in knowledge levels, lifestyle habits, and risk perceptions among various populations to enhance the translation of prevention knowledge into sustainable behavior change.

The evaluation of combined measures indicated that the triad of pulsed vector control, health education, and case isolation outperformed the pair of pulsed vector control and health education, albeit being slightly less effective than continuous low-intensity vector control. Whereas case isolation was a low-efficacy standalone measure, its incorporation into a combined strategy with pulsed vector control and health education enabled more effective outbreak containment at a reduced cost. Overall, this analysis demonstrated that case isolation served as a foundational intervention, vector control provided the strongest direct interruption of transmission, and health education highlighted the cost-effectiveness advantage of behavioral intervention. Thus, integrated strategies played an indispensable role in emergency outbreak response.

In addition, early initiation of interventions could enhance control effectiveness. Our data demonstrated that implementing health education measures (E = 50%, C = 60%) on day 30 of the epidemic could reduce cases by 55.37% compared with implementation on day 60. Similarly, low-intensity mosquito control initiated on day 30 reduced cases by 81.28% compared with initiation on day 60. In China, scholars have proposed multidimensional risk forecasting through the integration of healthcare diagnostic data, community health surveillance information, and population mobility trajectories [43]. However, Shangcheng District has not yet bridged institutional information silos across Cultural and Tourism, Customs, Commerce, and other governmental departments, clinical decision support systems lack automated travel-history alerts for patients from dengue-endemic regions. Consequently, border screening should be strengthened and improved, and multisectoral and regional cooperation mechanisms should be promoted for timely detection and follow-up surveillance. These measures demand prioritized implementation.

In summary, we propose a three-tiered synergistic dengue prevention system. The basic tier focuses on strict case isolation combined with intelligent surveillance and early warning to to reduce human-mosquito contact. The core tier targets the early epidemic stage, employing high-intensity continuous vector control (e.g., 10%-15% daily density reduction) to rapidly suppress mosquito density. The optimization tier utilizes coordinated interventions of pulsed vector control, health education, and case isolation to bridge the efficacy gaps of single measures while reducing costs. This three-tiered synergistic control system can effectively reduce the CC and shorten the DO, providing a replicable technical framework for DF control in high-density urban areas.

There are several limitations in our study. First, the effects of cross-reactive immunity among different dengue serotypes and antibody-dependent enhancement were not considered, which may bias transmission parameter estimation. Second, key parameters such as the *Aedes* mosquito lifespan, asymptomatic infection rate, behavior formation rates, and efficacy of mosquito prevention were derived from the literature rather than from local mosquito ecology monitoring, human serological surveys, and field trial data in the Shangcheng District. Third, since other districts in Hangzhou also experienced outbreaks during the same period, the inter-district transmission impacts were not taken into account, which might have led to an underestimation. Fourth, our model focused on short-term outbreak dynamics without incorporating regional climate variations or demographic resulting from births, natural deaths, or population migration. This limits the temporal applicability of the model. Fifth, the mosquito age-structure dimension was not considered. Variations in biting frequency, survival capacity, and viral susceptibility across different mosquito life stages might affect the accuracy of vector‑related transmission parameters, thereby introducing potential bias into the evaluation of intervention effectiveness. Future studies could be improved through multiple dimensions of research design. First, integrating viral gene sequencing technology would help elucidate the transmission dynamics differences among dengue virus serotypes. Second, standardized laboratory rearing experiments could be conducted to obtain basic biological data such as *Aedes* mosquito lifespan, while concurrently establishing a community-based cohort study to build a localized database of subclinical infection rates. Third, incorporating detailed vector surveillance data would clarify the impact of key characteristics such as mosquito age structure on the transmission process. At the same time, accounting for cross-regional epidemic linkages and population dynamics would further optimize the model.

## Conclusions

In conclusion, the SEIAR model effectively simulated both dengue fever epidemic dynamics and intervention effects in Shangcheng District. Model simulations demonstrated that the implemented control interventions during this outbreak were effective, preventing a majority of potential infections. Continuous vector control was the most effective single intervention, while the combination of pulse vector control, health education, and case isolation was more cost**-**effective.

## Data Availability

The datasets used and analyzed during this study and the numerical codes used to generate the outcomes of this paper are available from the corresponding author upon reasonable request.

## Abbreviations

DF: Dengue fever
DENV: Dengue virus
WHO: World Health Organization
SEIAR: Susceptible-exposed-infectious/asymptomatic-recovered
CC: Cumulative cases
DO: Duration of outbreak
DR: A daily mosquito density reduction
